# Ein geschlechterspezifischer Blick auf das gesundheitsbezogene Informationshandeln, seine Treiber und Barrieren: Ergebnisse einer Online-Befragung einer für die deutsche Bevölkerung stratifizierten Stichprobe

**DOI:** 10.1007/s00103-023-03757-6

**Published:** 2023-09-05

**Authors:** Elena Link, Eva Baumann, Christoph Aluttis

**Affiliations:** 1 Institut für Publizistik, Johannes Gutenberg-Universität Mainz, Jakob-Welder-Weg 12, 55128 Mainz, Deutschland; 2 Institut für Journalistik und Kommunikationsforschung, Hochschule für Musik, Theater und Medien Hannover, Hannover, Deutschland; 3 Referat Q6 – Erwachsene, Ältere, Frauen- und Männergesundheit, Chancengleichheit, Bundeszentrale für gesundheitliche Aufklärung, Köln, Deutschland

**Keywords:** Informationssuche, Informationsvermeidung, Ressourcen, Informationsquellen, Gesundheitskommunikation, Gender, Information seeking, Information avoidance, Resources, Information sources, Health communication, Gender

## Abstract

**Hintergrund:**

Vor dem Hintergrund der Geschlechterperspektive als relevante Ebene effektiver Gesundheitskommunikation wird das gesundheitsbezogene Informationshandeln fokussiert. Ergänzend zur Informationssuche wird die Informationsvermeidung einbezogen. Beide Formen des Informationshandelns werden geschlechterspezifisch beschrieben und sollen anhand verschiedener Ressourcen wie der *Gesundheit*, dem *Wohlbefinden*, dem *Empowerment*, dem *Involvement* und der *sozialen Unterstützung* erklärt werden.

**Methode:**

Es wurde eine Online-Befragung einer für die deutsche Bevölkerung stratifizierten Stichprobe (*N* = 3000) durchgeführt. Erfasst wurden die beiden Formen des Informationshandelns sowie ihre theoretisch abgeleiteten Prädiktoren. Neben deskriptiven Analysen dienen blockweise Regressionsanalysen dazu, geschlechterspezifisch die Prädiktoren der Suche und Vermeidung zu identifizieren.

**Ergebnisse:**

Die Informationssuche findet häufiger statt als die Informationsvermeidung. Beide unterscheiden sich nur in geringem Maß zwischen Männern und Frauen. Für Suche und Vermeidung zeigen sich die stärksten Zusammenhänge mit dem *Involvement* der Befragten. Für die Suche sind zudem auch Ressourcen des Bedrohungsmanagements bedeutsam, während die Vermeidung mit dem psychischen Wohlbefinden und der sozialen Unterstützung assoziiert ist. Geschlechterübergreifend zeigen sich ähnliche Assoziationsmuster.

**Diskussion:**

Geschlechterübergreifend zeigt sich, dass mittels gesundheitskommunikativer Maßnahmen das *Involvement* der Bevölkerung unterstützt und Ressourcen des Bedrohungsmanagements gestärkt werden müssen, um die Suche zu fördern und der Vermeidung entgegenzuwirken. Zudem wird deutlich, dass Vermeider*innen als eine zentrale Zielgruppe mehr Aufmerksamkeit erhalten sollten.

## Abstract (en)

**Background:**

The gender perspective is an essential level of effective health communication approaches. Against the background of the gender perspective as an essential level of effective health communication approaches, we focus on health-related information behaviors. Complementary to information seeking, information avoidance is included. Both forms of information behavior are described in gender-specific ways, and their predictors are identified using a resource-centered approach covering the individual’s *health*, *empowerment*, *involvement*, and *social support*.

**Method:**

An online survey of a sample stratified for the German population (*N* = 3000) was conducted. The two modes of information behavior as well as their theoretically derived predictors were assessed. In addition to descriptive analyses, blockwise regression analyses were used to identify gender-specific predictors of information seeking and avoidance.

**Results:**

Information seeking occurs more frequently than information avoidance. Both differ only slightly between men and women and showed the strongest correlations with the respondents’ involvement. For information seeking, coping resources are also a significant predictor, while information avoidance is associated with psychological well-being and social support. Similar patterns of association emerge across genders.

**Conclusion:**

Across genders, it is evident that health communication measures should strengthen the involvement of the population and their coping resources to promote information seeking and counteract information avoidance. Additionally, the findings revealed that avoiders should be more focused as one of the main target groups of health communication.

## Hintergrund

Männer und Frauen unterscheiden sich hinsichtlich ihrer biologischen Gesundheitsrisiken sowie ihrer Mortalität und Morbidität. Zusätzlich können auch von Geschlechteridentitäten beeinflusste Einstellungen zur Hilfs- und Informationssuche bei denselben Krankheiten zu unterschiedlichen Gesundheitsergebnissen von Frauen und Männern führen [1]. Beispielsweise können Normen Männer davon abhalten, bei körperlicher Krankheit und emotionaler Belastung medizinische Hilfe zu suchen oder Vorsorgeuntersuchungen in Anspruch zu nehmen [2–4], während Frauen aufgrund der Erwartung, dass sie fürsorglich sind, eher um relevante Unterstützung bitten oder solche Hilfe anbieten [2, 5].

Neben diesen unterschiedlichen gesundheitsrelevanten Verhaltensweisen zeigen sich auch Unterschiede im Informations- und Kommunikationshandeln zwischen den Geschlechtern. Der aktuelle Forschungsstand legt nahe, dass Frauen ein stärker ausgeprägtes Interesse an Gesundheitsinformationen haben und häufiger nach Gesundheitsinformationen suchen als Männer [6–11]. Somit ist die Geschlechterperspektive eine relevante Ebene für eine effektive Gesundheitskommunikation. Die Berücksichtigung entsprechender Unterschiede in der Gesundheitskommunikation scheint bedeutsam, um geschlechterspezifischen Bedürfnissen gerecht zu werden und einer Kluft zwischen den beiden Geschlechtern hinsichtlich ihres Umgangs mit Gesundheitsinformationen und der Gesundheitsversorgung entgegenzuwirken [12].

Der vorliegende Beitrag liefert eine solche geschlechterspezifische Perspektive auf das gesundheitsbezogene Informationshandeln und soll zu dessen Verständnis beitragen. Während bisherige Forschung oftmals nur die Häufigkeit der Zuwendung zu Gesundheitsinformationen betrachtet (insbesondere zu spezifischen Quellen wie dem Internet [9, 10]), ergänzt der vorliegende Beitrag diese Perspektive um die Vermeidung von Gesundheitsinformationen. Die *Suche nach Gesundheitsinformationen *beschreibt die aktive und zielgerichtete Zuwendung zu Informationen [13, 14]. Zu den Schlüsselmomenten dieses Prozesses zählt die Auswahl nützlicher Informationsquellen [15, 16]. Unter der Vielzahl von interpersonalen und medialen Quellen kann jeder und jede Einzelne in Abhängigkeit des eigenen Informationsbedürfnisses und der Quelleneigenschaften die passende Quelle situationsbedingt auswählen [15, 17–19]. Betrachtet man, welche medialen Quellen besonders häufig genutzt werden, kommt vor allem digitalen Informations- und Unterstützungsangeboten eine steigende Bedeutung zu [20, 21].

Obwohl in der Forschung bisher häufig die Informationssuche fokussiert wird, ist auch die *Vermeidung von Gesundheitsinformationen* ein alltägliches Kommunikationsphänomen [22–24]. Die Vermeidung ist keineswegs das Gegenteil der Informationssuche oder beschreibt ihr Ausbleiben, vielmehr handelt es sich ebenfalls um eine aktive und zielgerichtete Handlung [25, 26]. Die Informationsvermeidung zielt darauf ab, bewusst den Erwerb verfügbarer und persönlich relevanter, aber potenziell unerwünschter Informationen zu verhindern oder zeitlich hinauszuzögern [27]. Analog zur Suche legt eine Studie von Link [23] nahe, dass auch bei der Vermeidung verschiedene Quellen unterschieden werden sollten. Die Befragung im Zuge der COVID-19-Pandemie zeigte, dass interpersonale Quellen weniger stark vermieden werden als massenmediale Quellen [23]. Vor allem soziale Medien scheinen kritisch bewertet zu werden. Vor diesem Hintergrund werden in der vorliegenden Studie folgende Forschungsfragen (FF) untersucht:

FF 1: Was charakterisiert den Umgang mit Gesundheitsinformationen von Frauen und Männern?

Ermittelt werden soll zudem, welche Prädiktoren mit dem Informationshandeln von Frauen und Männern assoziiert sind. Die Erkenntnisse sollen Aufschluss über die Treiber und Barrieren des Informationshandelns geben und darüber, welche Prädiktoren somit förderlich bzw. hinderlich für das entsprechende Handeln sind. In Überblicksartikeln wird bisher zwischen personenbezogenen, situationalen und kontextspezifischen Prädiktoren unterschieden [1, 14, 28]. Im vorliegenden Artikel sollen personenbezogene Prädiktoren fokussiert werden. Geschlechterspezifisch sollen dabei das Alter, die Bildung und der Migrationshintergrund betrachtet werden, die laut aktuellen Forschungsergebnissen mit dem Informationshandeln zumindest in geringem Maße in Beziehung stehen [14]. Ergänzend wird ein ressourcenzentrierter Ansatz für die Auswahl der potenziell förderlichen oder hinderlichen Prädiktoren gewählt. Dem liegt die Annahme zugrunde, dass die Informationssuche und -vermeidung bestimmte Ressourcen für ihre Umsetzung benötigen. Ressourcen umfassen dabei nicht nur das Wissen oder die Kompetenzen eines Individuums, es können ebenso Haltungen, Persönlichkeitsmerkmale oder soziale Beziehungen sein.

Unterscheiden lassen sich verschiedene Ressourcen, die für das Informationshandeln relevant sind: *Gesundheit* im Sinne des körperlichen, seelischen und sozialen Wohlbefindens kann selbst als Ressource verstanden werden [29, 30]. Ebenso fungiert das individuelle *Empowerment* in Form des Wissens und der persönlich empfundenen Kontrolle als Ressource. Es wirkt sich darauf aus, inwiefern sich Individuen für den Erhalt ihrer Gesundheit engagieren, Verantwortung für die eigene Gesundheit tragen und selbstbestimmt handeln [31–33]. Zu individuellen Haltungen zählen auch die Motivation, sich mit Gesundheitsinformationen auseinanderzusetzen, und der Nutzen, der diesen Informationen zugeschrieben wird [34]. Diese beiden Bestandteile werden als Teil des *Involvements* mit der eigenen Gesundheit verstanden. Als weitere Form der empfundenen Wichtigkeit der Auseinandersetzung mit der eigenen Gesundheit sollen zudem zeitliche Ressourcen berücksichtigt werden.

Gerade für die Auseinandersetzung mit bedrohlichen Gesundheitsinformationen gilt es auch, *Ressourcen des Bedrohungsmanagements* zu berücksichtigen [35–38]. Es wird davon ausgegangen, dass diese Persönlichkeitsmerkmale Reaktionen auf Bedrohungen wie gesundheitliche Herausforderungen abpuffern und bei ihrer Bewältigung (*Coping*) helfen [37, 38]. Zu den relevanten Ressourcen zählen das Selbstwertgefühl, der Optimismus und die Tendenz zur spontanen Selbstbestätigung. Das Selbstwertgefühl ist eine Ressource, die auf dem Zugang zu positiven Aspekten des Selbst basiert und dadurch in der Lage ist, mit bedrohlichen Informationen umzugehen [27, 39]. Optimismus ist ein Persönlichkeitsmerkmal, das den Grad der positiven Zukunftserwartungen einer Person beschreibt. Ebenso kann die Tendenz zur spontanen Selbstbestätigung als ein Persönlichkeitsmerkmal oder eine Gewohnheit verstanden werden. Sie dient als Copingstrategie, indem sich ein Individuum als Reaktion auf eine Bedrohung auf ihre oder seine Werte oder Eigenschaften besinnt [37, 38, 40, 41]. Für alle *Ressourcen des Bedrohungsmanagements* wird angenommen, dass ein höheres Maß an Ressourcen mit mehr Suche und weniger Vermeidung verbunden ist [37, 38, 42]. Demnach reduzieren diese Ressourcen eine abwehrende Haltung gegenüber bedrohlichen Informationen [39, 40, 43] und Individuen sind motivierter, sich bedrohlichen Informationen zu stellen [39].

Zudem gilt es auch, soziale Ressourcen zu berücksichtigen. Die Verfügbarkeit *sozialer Unterstützung* senkt das Stressniveau des oder der Einzelnen und verringert die Auswirkungen ungünstiger Lebensbedingungen [27, 35, 44, 45]. Der Forschungsstand ist hierzu jedoch uneinheitlich. So konnten Emanuel et al. [42] nicht bestätigen, dass soziale Unterstützung das Vermeidungsverhalten verringert. Aus dem dargelegten Hintergrund resultieren die folgenden beiden FF:

FF 2: Welche der theoretisch abgeleiteten Ressourcen stellen Prädiktoren der gesundheitsbezogenen Informationssuche von Frauen und Männern dar?

FF 3: Welche der theoretisch abgeleiteten Ressourcen stellen Prädiktoren der gesundheitsbezogenen Informationsvermeidung von Frauen und Männern dar?

## Methode

Zur Beantwortung der FF wurde im Dezember 2021 eine Online-Befragung durchgeführt (*N* = 3000). Mittels eines Online-Access-Panels (Bilendi & respondi) wurde eine anhand der deutschen Bevölkerung nach Alter (18 bis 74 Jahre), Geschlecht, Bildung und Region stratifizierte Stichprobe rekrutiert. Die Befragten waren im Durchschnitt 46,13 Jahre alt (Standardabweichung (*SD*) = 15,36). 50,1 % fühlten sich dem weiblichen und 49,9 % dem männlichen Geschlecht zugehörig. 33,7 % hatten höchstens einen Hauptschulabschluss, 32,3 % haben einen Realschulabschluss und 34 % haben mindestens das Abitur. 15,4 % der Befragten hatten einen Migrationshintergrund. Die Befragten wurden zu Beginn der Umfrage umfassend über den Zweck und die Inhalte der Befragung aufgeklärt und um ihre informierte Einwilligung zur Teilnahme gebeten.

Das Erhebungsinstrument erfasste die Suche und die Vermeidung von Gesundheitsinformationen. Die Frequenz der Informationssuche wurde in Anlehnung an Mead et al. [46] erhoben. Auf einer 5‑stufigen Likert-Skala konnten die Befragten von 1 (nie) bis 5 (sehr häufig) angeben, wie oft sie sich gezielt über gesundheitliche und medizinische Themen informieren (Mittelwert (*M*) = 3,12; *SD* = 0,89). Zusätzlich wurde gefragt, wie häufig die Befragten 16 massenmediale und interpersonale Quellen frequentieren (Tab. 1). Die gezielte Vermeidung von Gesundheitsinformationen wurde mittels einer angepassten Version der Informational Opt-out Scale erfasst [23, 47, 48]. Mittels 7 Items wurde die Vermeidung verschiedener Informationsquellen abgefragt (Tab. 2). Die verwendete Skala reichte analog zur Suche von 1 (nie) bis 5 (sehr häufig). Nach erfolgreicher Reliabilitätsprüfung (α = 0,871) wurden die Items zu einem Mittelwertindex zusammengefasst (*M* = 2,62; *SD* = 1,00).

**Table 1:** 

Abhängige Variablen	Frauen (*n* = 1502) *M (SD)*	Männer (*n* = 1498) *M (SD)*	Gesamt (*N* = 3000) *M (SD)*	Unterschiede (F-Statistik)
Frequenz der Informationssuche***	*3,23 (0,87)*	*3,01 (0,90)*	*3,12 (0,86)*	*F (1, 2998)* *=* *45,22, p* *≤* *0,001, η*^*2*^ *=* *0,015*
Ärzt*innen und medizinisches Fachpersonal*	3,36 (1,13)	3,27 (1,19)	3,31 (1,16)	*F* (1, 2998) = 3,98, *p* = 0,046 *η*^*2*^ = 0,001
Suchmaschinen***	3,40 (1,22)	2,89 (1,25)	3,15 (1,26)	*F* (1, 2998) = 128,19, *p* ≤ 0,001, *η*^*2*^ = 0,041
Familienangehörige***	3,03 (1,18)	2,77 (1,17)	2,90 (1,18)	*F* (1, 2998) = 37,51, *p* ≤ 0,001, *η*^*2*^ = 0,012
Fachinformationen im Internet***	2,79 (1,25)	2,56 (1,21)	2,67 (1,24)	*F* (1, 2998) = 27,80, *p* ≤ 0,001, *η*^*2*^ = 0,009
Gesundheitsportale***	2,80 (1,26)	2,23 (1,20)	2,52 (1,26)	*F* (1, 2998) = 158,52, *p* ≤ 0,001, *η*^*2*^ = 0,050
Freund*innen***	2,64 (1,17)	2,28 (1,13)	2,46 (1,17)	*F* (1, 2998) = 74,15, *p* ≤ 0,001, *η*^*2*^ = 0,024
Fernsehen	2,38 (1,19)	2,45 (1,22)	2,41 (1,21)	*F* (1, 2998) = 2,42, *p* = 0,120 *η*^*2*^ = 0,001
Wikipedia und andere Online-Lexika***	2,50 (1,21)	2,32 (1,18)	2,41 (1,20)	*F* (1, 2998) = 17,47, *p* ≤ 0,001, *η*^*2*^ = 0,006
Staatl. Gesundheitsbehörden/gemeinnützige Organisationen*	2,26 (1,16)	2,18 (1,13)	2,22 (1,15)	*F* (1, 2998) = 3,96, *p* = 0,047 *η*^*2*^ = 0,001
Zeitschriften***	2,22 (1,18)	2,08 (1,11)	2,15 (1,15)	*F* (1, 2998) = 10,96, *p* ≤ 0,001, *η*^*2*^ = 0,004
Online-Communitys***	2,26 (1,17)	1,90 (1,06)	2,08 (1,13)	*F* (1, 2998) = 78,98, *p* ≤ 0,001, *η*^*2*^ = 0,026
Tages- und Wochenzeitungen	2,02 (1,09)	2,07 (1,11)	2,04 (1,10)	*F* (1, 2998) = 1,81, *p* = 0,179 *η*^*2*^ = 0,001
Kolleg*innen***	2,14 (1,19)	1,89 (1,05)	2,02 (1,13)	*F* (1, 2998) = 35,70, *p* ≤ 0,001, *η*^*2*^ = 0,012
Soziale Netzwerke***	1,97 (1,19)	1,76 (1,12)	1,86 (1,16)	*F* (1, 2998) = 24,93, *p* ≤ 0,001, *η*^*2*^ = 0,008
Hörfunk und Podcasts	1,64 (0,97)	1,68 (0,98)	1,66 (0,98)	*F* (1, 2998) = 1,38, *p* = 0,241, *η*^*2*^ = 0,000
Blogs***	1,59 (0,91)	1,48 (0,86)	1,54 (0,89)	*F* (1, 2998) = 12,18, *p* ≤ 0,001, *η*^*2*^ = 0,004

*N* = 3000, abhängige Variablen: Frequenz der allgemeinen Suche nach Gesundheitsinformationen sowie Suche nach Gesundheitsinformationen mittels ausgewählter Quellen gemessen auf einer Skala von 1 (nie) bis 5 (sehr häufig), signifikante Unterschiede zwischen Männern und Frauen sind wie folgt gekennzeichnet: *p < 0,05; **p < 0,01; ***p ≤ 0,001

**Table 2:** 

Abhängige Variable	Frauen (*n* = 1502) *M (SD)*	Männer (*n* = 1498) *M (SD)*	Gesamt (*N* = 3000) *M (SD)*	Unterschiede (F-Statistik)
Informationsvermeidung (Index)***	2,54 (0,98)	2,71 (1,02)	2,62 (1,00)	*F* (1, 2998) = 21,61, *p* ≤ 0,001, *η*^*2*^ = 0,007
Soziale Netzwerke***	2,93 (1,42)	3,14 (1,49)	3,03 (1,46)	*F* (1, 2998) = 16,62, *p* ≤ 0,001, *η*^*2*^ = 0,006
Hörfunk und Podcasts	2,68 (1,44)	2,78 (1,46)	2,73 (1,45)	*F* (1, 2998) = 3,56, *p* = 0,059 *η*^*2*^ = 0,001
Websites und Blogs***	2,58 (1,34)	2,81 (1,40)	2,69 (1,37)	*F* (1, 2998) = 20,86, *p* ≤ 0,001, *η*^*2*^ = 0,007
Fernsehberichte*	2,50 (1,25)	2,62 (1,30)	2,56 (1,27)	*F* (1, 2998) = 6,48 *p* = 0,011 *η*^*2*^ = 0,002
Zeitungs- und Zeitschriftenartikel**	2,46 (1,25)	2,58 (1,28)	2,52 (1,27)	*F* (1, 2998) = 7,36, *p* = 0,007 *η*^*2*^ = 0,002
Persönliche Gespräche mit Familienangehörigen und Freund*innen**	2,36 (1,18)	2,49 (1,22)	2,43 (1,20)	*F* (1, 2998) = 9,62, *p* = 0,002 *η*^*2*^ = 0,003
Private Nachrichten von Familienangehörigen und Freund*innen***	2,27 (1,23)	2,54 (1,33)	2,41 (1,29)	*F* (1, 2998) = 31,19, *p* ≤ 0,001, *η*^*2*^ = 0,010

*N* = 3000, abhängige Variablen: Frequenz der allgemeinen Vermeidung von Gesundheitsinformationen sowie Vermeidung von Gesundheitsinformationen in ausgewählten Quellen gemessen auf einer Skala von 1 (nie) bis 5 (sehr häufig), signifikante Unterschiede zwischen Männern und Frauen sind wie folgt gekennzeichnet: *p < 0,05; **p < 0,01; ***p ≤ 0,001

Als Prädiktoren der Informationssuche und -vermeidung wurde das Alter offen erfasst, 3 Bildungsgruppen mit höchstens Haupt- oder Volksschulabschluss, der mittleren Reife und mindestens (Fach‑)Abitur gebildet und für den Migrationshintergrund ermittelt, ob die Befragten oder mindestens ein Elternteil nicht in Deutschland geboren waren. Der subjektive Gesundheitszustand und das Vorhandensein einer chronischen Erkrankung wurde in Anlehnung an die Erhebung des Health Information National Trend Survey (HINTS) Germany [49] abgefragt. Die Befragten bewerteten ihren Gesundheitszustand auf einer 5‑stufigen Skala von 1 (sehr schlecht) bis 5 (sehr gut; *M* = 3,45; *SD* = 0,86) und konnten angeben, an welchen chronischen Erkrankungen (z. B. Hypertonie oder Arthritis und Rheuma) sie leiden. Für die vorliegende Auswertung wurde die Vielzahl der genannten chronischen Erkrankungen zusammengefasst. Es wurde nur einbezogen, ob die Befragten an mindestens einer Erkrankung leiden. Dies war bei einem Anteil von 61,7 % der Befragten der Fall. Das emotionale, psychische und soziale Wohlbefinden wurde anhand der Mental Health Continuum-Short Form [50] erfasst. Auf Basis der jeweils zufriedenstellenden Reliabilitätswerte pro Skala wurden 3 Mittelwertindizes berechnet (emotional: α = 0,885; *M* = 4,12; *SD* = 1,37, psychisch: α = 0,880; *M* = 3,40; *SD* = 1,25; sozial: α = 0,849; *M* = 4,06; *SD* = 1,23). Zur Messung von Wissen und Verstehen sowie der persönlichen Kontrolle als Dimensionen des *Empowerments* wurde die Gothenburg Young Persons Empowerment Scale adaptiert [33]. Für die eindimensionalen Subskalen wurde jeweils ein Mittelwertindex berechnet (Wissen: α = 0,756; *M* = 4,03; *SD* = 0,70; persönliche Kontrolle: α = 0,746; *M* = 3,88; *SD* = 0,75). Das *Involvement* wurde über 3 Items abgefragt, die darauf abzielen, die verfügbare Zeit (*M* = 2,32; *SD* =1,16), den zugeschriebenen Nutzen (*M* = 2,06; *SD* = 1,15) und die Motivation zur Auseinandersetzung mit Gesundheitsinformationen (*M* = 2,43; *SD* = 1,23) auf einer 5‑stufigen Likert-Skala abzufragen. Die Items waren dabei jeweils negativ formuliert (z. B. „Mir fehlt die Zeit, um mich mit Gesundheitsinformationen auseinanderzusetzen“). Die *Ressourcen des Bedrohungsmanagements* wurden in Überstimmung mit Taber et al. [37] gemessen. Die Skalen für den Selbstwert (α = 0,920; *M* = 3,79; *SD* = 0,88), die Tendenz zur spontanen Selbstbestätigung (α = 0,774; *M* = 3,13; *SD* = 1,04) und der Optimismus (α = 0,800; *M* = 3,38; *SD* = 0,94) wurden einer Reliabilitätsprüfung unterzogen und zu Mittelwertindizes zusammengefasst. Das Ausmaß der *sozialen Unterstützung* wurde in Anlehnung an Howell et al. [35] mittels 7 Items gemessen, die erneut auf einer Likert-Skala bewertet wurden (α = 0,908; *M* = 3,54; *SD* = 0,97).

### Datenanalyse

Zur Beantwortung von FF 1 wurden mittels deskriptiver Statistik die Verteilung der Informationssuche und Informationsvermeidung beschrieben. Zudem wurde zum Vergleich von Männern und Frauen jeweils eine Varianzanalyse pro Quelle berechnet. Zur Beantwortung von FF 2 und FF 3 wurden blockweise lineare Regressionsanalysen jeweils getrennt für Männer und Frauen durchgeführt. Der erste Block enthielt dabei die Variablen Alter, Bildung und Migrationshintergrund. Der zweite Block enthielt die Ressourcen.

## Ergebnisse

### Das Informationshandeln von Frauen und Männern (FF 1)

Die Befragten berichten, dass sie im Durchschnitt gelegentlich nach Gesundheitsinformationen suchen (*M* = 3,12; *SD* = 0,89). 4 % der Befragten geben an, dass sie noch nie nach Gesundheitsinformationen gesucht haben, während sich 31,3 % (sehr) häufig gezielt über gesundheitliche und medizinische Themen informieren. Besonders häufig aufgesuchte Informationsquellen stellen Ärzt*innen (*M* = 3,31; *SD* = 1,16), Suchmaschinen im Internet (*M* = 3,15; *SD* = 1,26) und Familienangehörige dar (*M* = 2,90; *SD* = 1,18; Tab. 1 für einen Überblick über alle genutzten Quellen). Betrachtet man die Häufigkeit der Informationssuche sowie die genutzten Quellen im Vergleich zwischen Männern und Frauen, so zeigen sich über alle Quellen hinweg zwar signifikante, aber relative geringe Unterschiede. Frauen suchen generell häufiger nach gesundheitlichen und medizinischen Themen und wenden sich einer höheren Anzahl von Quellen zu. Am stärksten zeigen sich Unterschiede bei der Nutzung von Suchmaschinen und Gesundheitsportalen. Beide Online-Angebote werden von Frauen häufiger herangezogen als von Männern. Im Gegensatz dazu finden sich keine signifikanten Unterschiede bei Fernsehen, Tages‑, Wochenzeitungen und Hörfunk.

In Bezug auf die gezielte Vermeidung von Gesundheitsinformationen geben 8,4 % der Befragten an, nie Informationen zu gesundheitsbezogenen Themen zu vermeiden. 30,3 % der Befragten weisen eine erhöhte Tendenz auf, Informationen über Gesundheitsthemen zu vermeiden. Über alle Quellen hinweg liegt der Mittelwert der Vermeidung über der Skalenmitte. Somit vermeiden die Befragten zumindest gelegentlich Gesundheitsinformationen (*M* = 2,62; *SD* = 1,00). Differenziert man dieses Verhalten anhand der vermiedenen Quellen, so zeigt sich, dass soziale Netzwerke am häufigsten vermieden werden (*M* = 3,03; *SD* = 1,46). Relativ häufig berichten die Befragten auch davon, dass sie bewusst Websites und Blogs (*M* = 2,69; *SD* = 1,37) ebenso wie Radiosendungen und Podcasts (*M* = 2,73; *SD* = 1,45) keine Aufmerksamkeit schenken (Tab. 2). Vergleichsweise seltener werden persönliche Gespräche mit Familienmitgliedern und Freund*innen (*M* = 2,43; *SD* = 1,20) sowie deren private Nachrichten (*M* = 2,41; *SD* = 1,29) vermieden. Unter Einbezug des Geschlechts der Befragten zeigt sich, dass Frauen generell seltener Gesundheitsinformationen vermeiden als Männer. Dieses Muster besteht dabei über alle Quellen hinweg. Allerdings sind die Unterschiede zwischen den Geschlechtern gering (Tab. 2).

### Prädiktoren der Auseinandersetzung mit Gesundheitsinformationen (FF 2/3)

Im Zuge der Beantwortung von FF 2 zu den Prädiktoren der Informationssuche zeigt sich, dass die Regressionsanalyse sowohl in der Stichprobe der Frauen als auch in der der Männer eine zufriedenstellende Erklärleistung aufweist. In der Stichprobe der Frauen können insgesamt 26,7 % der Varianz der Informationssuche erklärt werden, während es in der Stichprobe der Männer 28,1 % sind. Davon entfällt jeweils die Mehrheit (∆*R*^*2*^ = 25,0 % bzw. 27,8 %) auf die Ressourcen.

Betrachtet man die einzelnen Prädiktoren, zeigen sich in beiden Stichproben ähnliche Einflussmuster. Am einflussstärksten sind der zugeschriebene Nutzen (Frauen: *β* = −0,17; *p* ≤ 0,001; Männer: *β* *=* −0,19; *p* ≤ 0,001), die Motivation, sich Informationen anzueignen (Frauen: *β* = −0,29; *p* ≤ 0,001; Männer: *β* = −0,26; *p* ≤ 0,001), der Selbstwert (Frauen: *β* = −0,18*; p* ≤ 0,001; Männer: *β* = −0,25*; p* ≤ 0,001) und die Tendenz zur spontanen Selbstbestätigung (Frauen: *β* = 0,16*; p* ≤ 0,001; Männer: *β* = 0,12*; p* ≤ 0,001). Wird von den Befragten nur ein geringer Nutzen wahrgenommen und ist die Motivation zur Auseinandersetzung mit Gesundheitsinformationen gering, fungiert dies als Barriere der eigenen Suche. Ebenso ist auch der Selbstwert negativ mit der Informationssuche verbunden. Frauen und Männer mit einem höheren Selbstwert suchen seltener nach Gesundheitsinformationen. Im Gegensatz wirkt sich die Tendenz zur Selbstbestätigung positiv aus und führt dazu, dass sich die Befragten stärker Gesundheitsinformationen zuwenden. Ebenso förderlich scheint die Verfügbarkeit sozialer Unterstützung zu sein. Hier zeigt sich bei Männern (*β* = 0,12*; p* ≤ 0,001) ein stärkerer Zusammenhang als bei Frauen (*β* = 0,06*; p* = 0,025). Für beide Geschlechter steht zudem auch das psychische Wohlbefinden in einer positiven Beziehung zur Informationssuche (Frauen: *β* = 0,07*; p* = 0,044; Männer: *β* = 0,10*; p* = 0,01), wenn auch der Zusammenhang als eher schwach zu bewerten ist. Zudem findet sich eine schwache positive Beziehung zwischen dem Vorhandensein einer chronischen Erkrankung und der Informationssuche (Frauen: *β* = 0,06*, p* = 0,023; Männer: *β* = 0,07*; p* = 0,006).

Weitere signifikante Prädiktoren, die sich ausschließlich bei Frauen zeigen, sind die begrenzte Zeit (*β* = −0,06; *p* = 0,016) und der Bildungsgrad (*β* = 0,10; *p* ≤ 0,001). Während sich ein Zusammenhang zwischen der höheren Bildung der Frauen und der Informationssuche zeigt, führen begrenzte zeitliche Ressourcen zu einer tendenziell geringeren Suche (Tab. 3). Ausschließlich bei Männern zeigt sich, dass das emotionale Wohlbefinden (*β* = −0,09; *p* = 0,039) und der Optimismus (*β* = 0,09; *p* = 0,009) signifikant, wenn auch nur in einem geringen Ausmaß mit der Informationssuche verbunden sind (Tab. 3). Ein stärker ausgeprägtes emotionales Wohlbefinden der Männer ist tendenziell mit einer geringeren Suche assoziiert, während ein höher ausgeprägter Optimismus mit einer häufigeren Suche in Beziehung steht.

**Table 3:** 

	Informationssuche				Informationsvermeidung			
	Frauen		Männer		Frauen		Männer	
Prädiktoren	∆*R*^*2*^	*β*	∆*R*^*2*^	*β*	∆*R*^*2*^	*β*	∆*R*^*2*^	*β*
**Block 1: personenbezogene Merkmale**	0,017***		0,003*		0,016***		0,013***	
Alter	**–**	0,03	**–**	0,03	**–**	0,09**	**–**	0,08**
Bildung		0,10***		0,02		0,00		−0,04
Migration		−0,01		0,02		−0,01		−0,02
**Block 2: Ressourcen**	0,250***		0,278***		0,172***		0,186***	
*Gesundheit*								
Subjektiver Gesundheitszustand	**–**	−0,05	**–**	−0,03	**–**	−0,01	**–**	−0,08**
Vorhandensein einer chronischen Erkrankung		0,06*		0,07**		0,01		−0,02
Emotionales Wohlbefinden		−0,09		−0,09*		0,05		0,10*
Psychisches Wohlbefinden		0,07*		0,10**		−0,15***		−0,10**
Soziales Wohlbefinden		0,09		0,05		0,11*		−0,03
*Empowerment*								
Wissen und Verstehen	**–**	0,04	**–**	0,04	**–**	0,02	**–**	0,02
Persönliche Kontrolle		−0,04		0,04		−0,03		−0,01
*Involvement*								
Fehlende Zeit	**–**	−0,06*	**–**	−0,03	**–**	−0,02	**–**	0,13***
Geringer zugeschr. Nutzen		−0,17***		−0,19***		0,26***		0,19***
Geringe Motivation		−0,29***		−0,26***		0,19***		0,13***
*Ressourcen des Bedrohungsmanagements*								
Selbstwert	–	−0,18***	–	−0,25***	–	−0,10**	–	0,05
Tendenz zur spontanen Selbstbestätigung		0,16***		0,12***		0,01		−0,05
Optimismus		0,05		0,09**		0,10**		−0,03
*Soziale Ressourcen*								
Soziale Unterstützung	**–**	0,06*	**–**	0,12***	**–**	−0,10***	**–**	−0,14***
**Korr. *R***^***2***^	0,267***		0,281***		0,188***		0,199*	

*N* *=* *3000*; Ergebnisse von 2 blockweisen linearen Regressionsanalysen; abhängige Variablen: Frequenz der Suche sowie der Vermeidung von Gesundheitsinformationen jeweils gemessen auf einer Skala von 1 (nie) bis 5 (sehr häufig); unabhängige Variablen: erster Block: personenbezogene Merkmale, zweiter Bock: physischer und psychischer Gesundheitszustand; dritter Block: Barrieren und Treiber des Informationshandelns

*p ≤ 0,05; **p ≤ 0,01; ***p ≤ 0,001

In beiden Stichproben finden sich keine signifikanten Assoziationen der Informationssuche mit dem subjektiven Gesundheitszustand, dem sozialen Wohlbefinden, den beiden Dimensionen des *Empowerments*, dem Alter der Befragten und ihrem Migrationshintergrund (Tab. 3).

FF 3 bezieht sich auf die Informationsvermeidung. Hier zeigt sich eine im Vergleich zur Suche etwas geringere Erklärleistung der einbezogenen Prädiktoren. Insgesamt können 18,8 % der Varianz der Informationsvermeidung in der Stichprobe der Frauen und 19,9 % der Varianz in der Stichprobe der Männer erklärt werden (Tab. 3). Erneut liefern dabei die Ressourcen den stärksten Erklärbeitrag (∆*R*^*2*^ = 17,2 bzw. 18,6 %). Analog zur Suche bestätigt sich, dass im Vergleich der Geschlechter ähnliche Einflussmuster bestehen. Die stärksten Zusammenhänge finden sich erneut für den als gering wahrgenommenen Nutzen (Frauen: *β* = 0,26; *p* ≤ 0,001; Männer: *β* *=* 0,19; *p* ≤ 0,001) und die geringe Motivation (Frauen: *β* = 0,19; *p* ≤ 0,001; Männer: *β* *=* 0,13; *p* ≤ 0,001). Die Vermeidung von Gesundheitsinformationen erfolgt häufiger, wenn der Nutzen der Gesundheitsinformationen nicht erkannt wird und eine geringere Motivation zur Auseinandersetzung besteht. Ein Treiber der Vermeidung ist dagegen das psychische Wohlbefinden (Frauen: *β* = −0,15; *p* ≤ 0,001; Männer: *β* *=* −0,10; *p* = 0,014). Ist dieses eher gering ausgeprägt, fällt die Vermeidung von Gesundheitsinformationen in beiden Teilstichproben häufiger aus. Zudem ist die Vermeidung häufiger, wenn die befragten Männer und Frauen eine geringe soziale Unterstützung ihres Umfeldes wahrnehmen (Frauen: *β* = −0,10; *p* ≤ 0,001; Männer: *β* *=* −0,14; *p* ≤ 0,001). Ebenso steigt die Vermeidung von Gesundheitsinformationen tendenziell mit zunehmendem Alter der Befragten (Frauen: *β* = 0,09; *p* = 0,006; Männer: *β* = 0,08; *p* = 0,009).

Ausschließlich bei Frauen zeigt sich zudem, dass das soziale Wohlbefinden (*β* = 0,11; *p* = 0,041) und der Optimismus (*β* = 0,10; *p* = 0,006) positiv mit der Vermeidung assoziiert sind, während der Selbstwert (*β* = −0,10; *p* = 0,009) in einer negativen Beziehung zur Vermeidung von Gesundheitsinformationen steht (Tab. 3). Frauen, die über ein höheres soziales Wohlbefinden verfügen, generell optimistischer sind, aber auch ein geringeren Selbstwert besitzen, vermeiden somit häufiger Gesundheitsinformationen. In der Stichprobe der Männer sind das emotionale Wohlbefinden (*β* = 0,10; *p* = 0,041) und begrenzte zeitliche Ressourcen (*β* = 0,13; *p* ≤ 0,001) positiv mit der Vermeidung verbunden, während der subjektive Gesundheitszustand (*β* = −0,08; *p* = 0,007) schwach negativ mit der Vermeidung assoziiert zu sein scheint (Tab. 3). Männer vermeiden somit häufiger Informationen über ihre Gesundheit, wenn sie ein hohes emotionales Wohlbefinden besitzen, sie ihren Gesundheitszustand tendenziell schlechter wahrnehmen und ihnen zeitliche Ressourcen fehlen.

Im Gegensatz dazu zeigen sich keine signifikanten Zusammenhänge zwischen der Bildung, dem Migrationshintergrund, dem Vorhandensein chronischer Erkrankungen, beiden Dimensionen des *Empowerments *sowie der Tendenz zur spontanen Selbstbestätigung mit der Informationsvermeidung (Tab. 3).

## Diskussion

Der vorliegende Beitrag nimmt eine geschlechterspezifische Perspektive auf das Informationshandeln ein und erweitert den bestehenden Forschungsstand, indem die Suche und die Vermeidung fokussiert werden. Die gewonnenen Erkenntnisse zeigen, dass die gesundheitsbezogene Informationssuche verbreiteter ist als die Informationsvermeidung. Im Einklang mit bestehenden Forschungsergebnissen [19] zeigt sich, dass am häufigsten Ärzt*innen, Suchmaschinen und Familienangehörige frequentiert werden. Am häufigsten vermieden werden vor allem soziale Netzwerke, Websites und Blogs, während der Austausch mit dem privaten Umfeld vergleichsweise selten vermieden wird [23].

Sowohl für die Suche als auch für die Vermeidung zeigen sich nur geringe geschlechterspezifische Unterschiede. Tendenziell suchen Frauen häufiger und vermeiden seltener als Männer [9–11]. Aus diesen Erkenntnissen können wichtige Implikationen für die Gesundheitskommunikation abgeleitet werden. Zunächst untermauern die Erkenntnisse, welche Settings für die Vermittlung von Gesundheitsinformationen besonders geeignet scheinen. Neben Praxen von Ärzt*innen ist hier auch das Internet als Kanal für Gesundheitsinformationen hervorzuheben [20, 21]. Vor allem Frauen nehmen ihre Suche nach Gesundheitsthemen vor allem im Internet vor und können hier jeweils nicht nur mit Blick auf die eigene Gesundheit, sondern auch stellvertretend als Mittlerinnen in ihrem sozialen Umfeld adressiert werden. Zudem zeigt die Studie in Übereinstimmung mit dem aktuellen Forschungsstand [23, 24], dass fast jede dritte Person (30,3 %) eine erhöhte Tendenz zur Vermeidung von Gesundheitsinformation besitzt. Dies ist eine Herausforderung für die Prävention und Gesundheitsförderung, weil entsprechende Personen weniger wahrscheinlich von Gesundheitsinformationen erreicht werden, ihre Risikowahrnehmung verzerrt sein kann, gewünschte Änderungen ihrer gesundheitsbezogenen Einstellungen und Verhaltensweisen ausbleiben oder die Diagnose von Erkrankungen verspätet erfolgt. Dies kann folgenreich für die Gesundheit des oder der Einzelnen sowie das Gesundheitssystem sein. Um entsprechenden negativen Konsequenzen entgegenzuwirken, braucht es ein besseres Verständnis der Vermeidung ebenso wie die Entwicklung von Kommunikationsstrategien, die sich bewusst an diese schwer erreichbare Personengruppe richten. Zu solchen Strategien können die Ansprache des sozialen Umfeldes oder der Einsatz selbstbestätigender Botschaften zählen [38, 40, 41].

Mit Blick auf die Prädiktoren des Informationshandelns kann die vorliegende Studie aufzeigen, dass die Suche durch den ressourcenbezogenen Ansatz besser erklärt werden kann als die Vermeidung von Gesundheitsinformationen. Zudem fallen die Assoziationen teilweise eher gering aus. Dies verweist auf den weiteren Forschungsbedarf zur Identifikation weiterer Prädiktoren. Bei Frauen und Männern zeigen sich zudem sehr ähnliche Assoziationsmuster, wenn auch einzelne Unterschiede bestehen.

Vor allem das *Involvement* der befragten Männer und Frauen scheint für ihr Informationshandeln wichtig [21], was verdeutlicht, dass das Bewusstsein für die Bedeutung des Informationshandelns gefördert werden muss. Ebenso scheint das psychische und emotionale Wohlbefinden dafür zentral, inwiefern sich jemand in der Lage sieht, sich mit Gesundheitsinformationen auseinanderzusetzen. In diesem Kontext ist vor allem darauf hinzuweisen, dass die Suche keineswegs immer als positiv und die Vermeidung nur als negativ zu bewerten ist. Die Suche kann zur Überforderung führen, während die Vermeidung auch als *Coping*strategie fungiert und dabei hilft, positive emotionale Zustände aufrechtzuerhalten [25]. Dies scheint tendenziell etwas stärker bei Männern als bei Frauen ausgeprägt zu sein. Ausschließlich unter Männern ist das emotionale Wohlbefinden damit verbunden, dass weniger Informationen gesucht und mehr Informationen vermieden werden. Dieses geschlechterspezifische Muster kann ein Ansatzpunkt für weitere Forschung sein. Das Muster steht im Einklang damit, dass Männer häufiger Gesundheitsinformationen vermeiden, wenn sie ihren Gesundheitszustand tendenziell als schlechter wahrnehmen. Da vor allem dann Gesundheitsinformationen besonders bedeutsam sind, gilt es, Ängste als Barriere einer Auseinandersetzung mit Gesundheitsinformationen abzubauen. Ob Informationen gesucht oder vermieden werden, ist zudem auch von *Ressourcen des Bedrohungsmanagements* abhängig. Während ein höherer Selbstwert mit einem geringeren Informationshandeln verbunden ist, fördert der Optimismus Suche und Vermeidung. Die Tendenz zur spontanen Selbstbestätigung, indem man sich beispielsweise auf die eigenen Stärken besinnt, kann zur Suche beitragen. Die spontane Selbstbestätigung kann sowohl als Strategie erlernt, aber auch in Botschaften integriert werden, um die Auseinandersetzung mit Informationen zu erleichtern [38, 40, 41].

Die *soziale Unterstützung* scheint förderlich für die Suche und steht der Vermeidung entgegen. Diese Assoziation ist bei Männern stärker ausgeprägt als bei Frauen. Somit unterstreicht die Erkenntnis Potenziale des Einbezugs des sozialen Umfeldes in gesundheitskommunikative Maßnahmen. Speziell bei Frauen scheint auch eine formal niedrige Bildung eine Barriere der aktiven Informationssuche darzustellen und verweist somit auf eine spezielle Subgruppe, die besondere Aufmerksamkeit benötigt.

Für die Interpretation der Erkenntnisse müssen auch Limitationen berücksichtigt werden. Zunächst soll mit Blick auf die geringen geschlechterspezifischen Unterschiede darauf verwiesen werden, dass die Studie das Geschlecht nur dichotom abbildet, Geschlechteridentitäten nicht einbezieht [6–8] und nur unzureichend intersektional die Kombination verschiedener Personenmerkmale berücksichtigt. Mit Blick auf das beschriebene Informationshandeln und die geäußerten Quellenpräferenzen ist zudem zu hinterfragen, inwiefern die Befragten die einzelnen genutzten Quellen adäquat erinnern und einordnen können und inwiefern dieses Handeln kurz- und langfristig durch die COVID-19-Pandemie beeinflusst wurde. Während die Präferenzen für Informationsquellen relativ stabil sind [19, 21], kann davon ausgegangen werden, dass die Pandemie vor allem die Vermeidung von Gesundheitsinformationen aufgrund ihrer Omnipräsenz und einer zunehmenden Ermüdung der Bevölkerung verstärkt hat.

Zusammenfassend soll festgehalten werden, dass sich nur geringe geschlechterspezifische Formen des Informationshandelns zeigen. So suchen Frauen häufiger nach Gesundheitsinformationen. Zu den Personen, die Gesundheitsinformationen vermeiden und die anhand ihrer Prävalenz auch eine bedeutende Zielgruppe der Gesundheitskommunikation darstellen sollten, gehören tendenziell mehr Männer. Auch die Prädiktoren zeigen nur einzelne geschlechterspezifische Assoziationsmuster. Besonders bedeutsam als Barrieren und Treiber des Informationshandelns gilt es, geschlechterübergreifend das *Involvement* zu fördern und *Ressourcen des Bedrohungsmanagements* zu stärken.
